# Knowledge, Attitudes and Practices on Antimicrobial Usage and Resistance Among Broiler and Layer Poultry Farmers in Bangladesh: Lessons for Future Improvement

**DOI:** 10.1002/vms3.70776

**Published:** 2026-01-14

**Authors:** Zohir Raihan, Md. Nasir Uddin, Md. Shamsul Islam, Suman Das Gupta, M. Sawkat Anwer, Subir Sarker, Md. Hakimul Haque

**Affiliations:** ^1^ Department of Veterinary and Animal Sciences Faculty of Veterinary and Animal Sciences Rajshahi University Rajshahi Bangladesh; ^2^ School of Agricultural, Environmental and Veterinary Sciences Faculty of Science and Health Charles Sturt University Wagga Wagga New South Wales Australia; ^3^ Gulbali Institute Charles Sturt University Wagga Wagga New South Wales Australia; ^4^ Department of Biomedical Sciences Cummings School of Veterinary Medicine Tufts University North Grafton Massachusetts USA; ^5^ Biomedical Sciences & Molecular Biology College of Medicine and Dentistry James Cook University Townsville Queensland Australia; ^6^ Australian Institute of Tropical Health and Medicine James Cook University Townsville Queensland Australia; ^7^ Australian Institute for Bioengineering and Nanotechnology (AIBN) The University of Queensland Brisbane Queensland Australia

**Keywords:** AMR, AMU, antimicrobial, KAP, livestock, poultry farmers, poultry farms

## Abstract

**Background:**

Antimicrobial resistance (AMR) is a critical global health concern, closely linked to the excessive and unregulated use of antimicrobials in livestock production systems, particularly in poultry farming. In Bangladesh, where poultry serves as a key source of animal protein, the misuse of antimicrobials contributes to the rapid emergence and spread of AMR, endangering animal, environmental and human health. Poultry farmers play a vital role in mitigating AMR through responsible antimicrobial usage (AMU), underscoring the urgent need for targeted educational interventions and strengthened regulatory frameworks to promote prudent AMU practices.

**Methods:**

This cross‐sectional study assessed the knowledge, attitudes and practices (KAP) of poultry farmers regarding AMU across three districts in Bangladesh: Bogura, Rajshahi and Munshiganj. Data were collected from 294 poultry farmers through face‐to‐face interviews using a structured, pre‐validated questionnaire. KAP was classified using descriptive statistics and the chi‐square tests (*p* < 0.05).

**Results:**

A majority of farmers (98.64%) reported us antimicrobials; however, only 50.34% obtained veterinary prescriptions. In addition, 73.13% were unaware of authorized prescribers, and 91.16% had no prior knowledge of AMR. Antimicrobials were frequently used during the brooding phase (61.90%) and as growth promoters (39.46%). A significant proportion of farmers (65.31%) believed antimicrobials could be used without veterinary advice, and 80.61% held misconceptions about their efficacy against viral infections. Furthermore, about 48.98% purchased these antimicrobials from local pharmacies without prior consultation with a veterinarian. The most commonly used antimicrobials were ciprofloxacin (58.84%), levofloxacin (43.20%), colistin (39.12%), amoxicillin (36.39%), doxycycline (36.39%) and tylosin (30.95%).

**Conclusions:**

The widespread lack of knowledge and inappropriate attitudes toward AMU among poultry farmers is a significant driver of AMR. Addressing this issue necessitates comprehensive educational programs to enhance awareness, stricter enforcement of veterinary regulations to ensure responsible antimicrobial use and the establishment of robust AMU surveillance systems for continuous monitoring and assessment.

## Graphical abstract

This study highlights widespread antimicrobial misuse among Bangladeshi poultry farmers, driven by low AMR awareness (91.16%), non‐prescription purchases (48.98%), and reliance on dealers (reliance on dealers). Targeted education, stricter regulations, and surveillance are crucial to foster responsible practices and mitigate AMR risks.

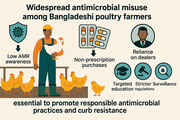

## 1 Introduction

In recent decades, poultry meat and egg production in South Asia, particularly Bangladesh, has witnessed unprecedented growth, becoming a cornerstone of the region's agricultural economy (M. S. Hassan 2022). The poultry sector is now the country's second‐largest industry after the ready‐made garment (RMG) sector, driven by intensive farming systems to meet the rising demand for affordable animal protein (M. M. Hassan et al. 2021). Beyond ensuring food security, poultry farming serves as a vital source of income and economic stability for rural communities. Recent reports indicate that poultry accounted for a substantial portion of total livestock production, with 385.704 million birds produced from the total livestock population of 442.847 million in 2022–2023 (DLS 2023). With approximately 150,000 commercial poultry farms, both local and international businesses contribute significantly to this sector (DoLS Department of Livestock Service 2014). Currently, poultry products supply 37% of the Bangladesh's total animal protein intake (Hamid et al. 2017).

Despite significant advancements in poultry farming, the misuse and overuse of antimicrobials have emerged as major public health concerns, accelerating the development of antimicrobial resistance (AMR) (M. M. Hassan 2020). AMR poses a severe threat to global health, complicating the treatment of infectious diseases in both humans and animals (Go'chez et al. 2019). In poultry farming, antimicrobials are widely used for disease prevention, growth promotion and therapeutic purposes often without veterinary oversight contributing to enhance productivity but also driving the selection and dissemination of resistant pathogens (M. M. Hassan et al. 2014; Sarwar et al. 2018). The widespread, unregulated antimicrobial usage (AMU), coupled with limited knowledge and inappropriate practices, has further exacerbated the AMR crisis (Ferri et al. 2017).

In Bangladesh, AMU in food‐producing animals remains largely unregulated, with farmers frequently relying on self‐prescription or guidance from unqualified sources. This unstructured and often indiscriminate approach is a key driver of AMR, contributing to animal health issues, environmental contamination and increased risks to human well‐being (World Health Organization 2021). Recognizing this growing concern, the Bangladesh government has introduced the National Strategic Plan (NSP) and National Action Plan (NAP) for Antimicrobial Resistance (2021–2026), aligned with the Global Action Plan (GAP) on AMR (Jhora 2021). However, effective implementation of these policies requires coordinated efforts from all stakeholders, including farmers, veterinarians and policymakers.

By 2050, AMR is projected to cause 10 million deaths annually, with low‐ and middle‐income countries (LMICs) in Africa and Asia expected to bear the greatest burden (deKraker et al. 2016; Hofer 2019). The economic implications are equally alarming, with drug‐resistant infections expected to reduce the global gross domestic product (GDP) by 3.8% per year. In poultry farming, excessive antibiotic use accelerates the spread of resistance genes and antimicrobial residues through the food chain, soil and environment, further amplifying the development of resistance (Al Masud et al. 2020; Hedman et al. 2020; Kousar et al. 2021; Om and McLaws 2016). Efforts to combat AMR in the poultry sector must address critical gaps in farmers’ knowledge, attitudes and practices regarding AMU. However, the extent of these deficiencies remains poorly understood. Unlike previous studies (Imam et al. 2020; M. M. Hassan et al. 2021), our research: (i) Includes both broiler and layer farmers across three diverse districts (Bogura, Rajshahi and Munshiganj), providing broader geographical representation; (ii) offers a comparative analysis between broiler and layer farm types, revealing significant differences in AMU patterns (e.g., higher reliance on dealers among broiler farmers); (iii) assesses specific antimicrobial agents used and their frequency, including critically important ones like colistin; and (iv) provides policy‐relevant recommendations aligned with Bangladesh's NAP on AMR (2021–2026), including actionable strategies for small‐scale farmers. These aspects highlight the unique contribution of our study in informing targeted interventions in Bangladesh's poultry sector. Strengthening antimicrobial stewardship requires targeted educational programs, awareness campaigns and stringent enforcement of veterinary regulations. In addition, continuous surveillance and research on AMU trends are essential for shaping effective policies and interventions (WHO 2016). Addressing AMR demands a multi‐sectoral approach, integrating technical guidance, legal enforcement and community engagement.

This study aims to assess the knowledge, attitudes and practices (KAP) of commercial poultry farmers in Bangladesh regarding AMU and AMR. By generating evidence‐based insights, the study seeks to inform policy recommendations aligned with the NAP on AMR, contributing to the development of sustainable strategies for resistance management and the promotion of responsible antimicrobial use (AMU) in the poultry industry.

## 2 Materials and Methods

### 2.1 Ethical Consideration

The ethical approval was received from the Institute of Biological Sciences (IBScs), University of Rajshahi, Bangladesh (Memo No. 56/321/IAMEBBC/IBScs). Prior to data collection, written informed consent was obtained from all participants. The research objectives were clearly explained to each participant, emphasizing their right to decline or withdraw from the study at any stage without any consequences. Strict measures were employed to ensure the confidentiality of the information provided and maintain all participants' anonymity throughout the research process.

### 2.2 Study Design and Population

This cross‐sectional study was conducted over six months, from September 2022 to February 2023, targeting poultry farmers actively engaged in commercial production. The study included a total of 294 participants from Bogura, Rajshahi and Munshiganj districts in Bangladesh, representing both layer and broiler farming operations. Farmers were randomly selected to ensure a diverse and representative sample of the poultry farming community. Participants had to meet the following eligibility criteria to participate in the study: (i) Ongoing operations in broiler or layer poultry farming, (ii) Respondents manage daily decisions in farm management, and (iii) the ability to provide accurate information regarding their farming practices. Participation in the study was entirely voluntary, with no financial or material incentives were offered to encourage participation. Certain farmers were excluded from the study based on the following exclusion criteria: (i) Those who had ceased poultry farming or did not have any chickens at the time of the survey; (ii) those unable to provide relevant or reliable information about their farm practices; and (iii) farms involved in poultry operations outside the scope of broiler or layer production. This approach ensured the collection of reliable data on AMU practices and resistance awareness among commercial poultry farmers while maintaining methodological rigor and adherence to ethical standards.

### 2.3 Study Area

**FIGURE 1:**
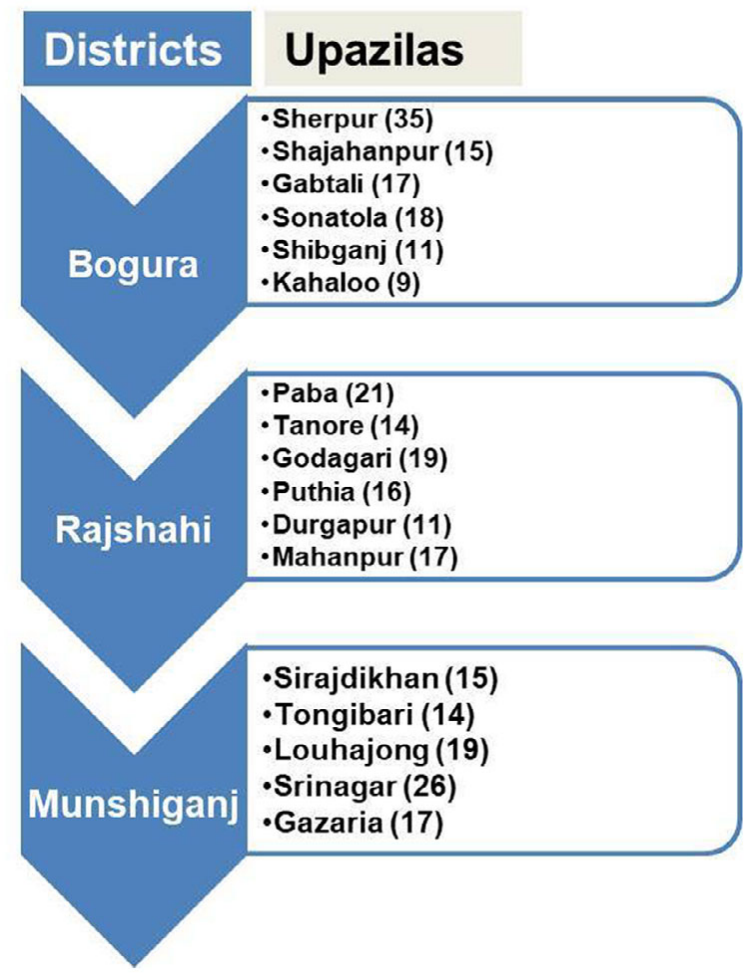
The Sampling procedure flowchart displays the number of farms visited across various upazilas in the districts of Bogura, Rajshahi and Munshiganj.

The study was conducted across 17 upazilas in three districts of Bangladesh, as detailed in Figure 1. Two of these districts, Bogura and Rajshahi, are situated in the northwest region of Bangladesh under the Rajshahi Division, sharing a southern border with the Indian state of West Bengal. The third district, Munshiganj, is positioned in central Bangladesh within the Dhaka Division, approximately 58 kilometres from Dhaka, the nation's capital. The Rajshahi Division lies between 23°48′ and 25°16′ north latitude and 88°01′ and 89°48′ east longitude, while Munshiganj is located between 23°23′N to 23°38′N latitude and 90°10′N to 90°53′E longitude. Geographically, the Rajshahi division is bordered by Naogaon, Joypurhat and Gaibandha districts to the north; the Padma River, Kushtia, Natore, Shirajganj districts, and parts of West Bengal to the south; the Jamuna River to the east; and Nawabganj district to the west. Munshiganj serves as a key poultry supply hub for Dhaka, while Bogura and Rajshahi districts play a central role in poultry production for Rajshahi city and surrounding areas. In recent years, these districts have seen a notable increase in the number of poultry farms, making them pivotal areas for poultry production in Bangladesh. However, data on AMU in these regions remain limited. Given the significant expansion of poultry farming and the potential risks associated with antibiotics misuse, these three districts were selected as the focus areas for this study to gain insights into AMU practices and AMR awareness among poultry farmers.

### 2.4 Sample Size Determination

The required sample size for this study was determined using the following standard sample size calculation formula:$\;n=\frac{z^{2}pq}{d^{2}}$

Substituting the values into the formula:

$$n=\frac{1.96^{2}\times 0.5\times 0.5}{0.07^{2}}=196$$

Here, *z* represents the *z*‐score corresponding to a 5% level of significance, which is 1.96, and *d* is the satisfactory margin of error set at 7% (0.07). The proportion of the target population possessing the characteristic of interest (*p*) was assumed to be 50% (0.5), as there was no prior study in this specific cohort within the study area. Consequently, the complement of *p*, denoted as *q*, is (1−*p*) or 50% (0.5). Based on this calculation, the minimum required sample size was 196 participants. However, to improve the statistical power and robustness of the study, a total of 294 commercial poultry farmers were recruited.

### 2.5 Data Collection Tools and Techniques

Data were collected through face‐to‐face interviews using a semi‐structured questionnaire designed to capture comprehensive information on AMU and AMR awareness among poultry farmers. The questionnaire consisted of six distinct sections covering key aspects: (i) Socio‐demographic characteristics and poultry farming details, (ii) knowledge of owners towards AMU and AMR in poultry farming, (iii) attitudes of farmers regarding AMU and AMR reflects beliefs about resistance risks, (iv) practices related to AMU, (v) sources of information and patterns of antibiotic use in poultry and (vi) commonly used antimicrobials in poultry production. To ensure the reliability and validity of the semi‐structured questionnaire used in this study, a rigorous validation process was conducted. The questionnaire was pre‐validated by a panel of five subject matter experts, comprising three veterinarians with expertise in poultry health and AMU and two public health specialists with experience in AMR research. The Item‐Objective Congruence (IOC) index, as described by Rovinelli and Hambleton (1977), was employed to assess content validity (Rovinelli and Hambleton 1977). Each expert rated the congruence of each questionnaire item with its intended objective on a scale of −1 (not congruent), 0 (uncertain) or +1 (congruent). The IOC index was calculated using the formula:

$$\mathrm{IOC}\;=\frac{\left(\Sigma R\;-\;N\right)}{\left(N*M\;-\;1\right)}$$

where Σ*R* is the sum of ratings for a specific item–objective pair, *N* is the number of experts (5) and *M* is the number of objectives evaluated.

All items achieved an IOC score of ≥0.70, with an average IOC score of 0.82 across all items, indicating strong alignment with the study objectives of assessing knowledge, attitudes, and practices (KAP) related to AMU and AMR. In addition, internal consistency was evaluated using Cronbach's alpha, yielding a value of 0.78, which confirms the questionnaire's reliability for consistent measurement across respondents. The questionnaire was also pretested on a subset of 20 poultry farmers to ensure clarity, cultural relevance and ease of comprehension, with minor revisions made to improve question phrasing based on feedback. These validation steps ensured the instrument's accuracy and suitability for capturing reliable data in the context of Bangladesh's poultry farming sector. To facilitate accurate responses and minimize potential misinterpretation of questions, interviews were conducted in the local language.

### 2.6 Socio‐Demographic Characteristics and Poultry Farming Information

During the interviews, participants were asked questions regarding their socio‐demographic details and poultry farming practices. These questions covered various aspects, including the respondent's age, gender, educational background, family income, primary source of household income, the number and types of chickens raised, the production system employed and participants’ level of experience in poultry farming.

### 2.7 Knowledge of AMU in Poultry Farms

Farmers' knowledge regarding AMU was assessed using ten questions, adapted from a previously conducted study (Alhaji et al. 2018).

### 2.8 Assessment of Poultry Farmers' Attitudes toward AMU and AMR

The attitudes of poultry farmers regarding AMU and AMR were evaluated through a structured questionnaire consisting of nine targeted questions.

### 2.9 Practices Related to AMU in Poultry Farming

Practices concerning AMU were evaluated using 6 questions. These questions were derived from prior studies (Alhaji et al. 2018; Caudell et al. 2020; McKernan et al. 2021).

### 2.10 Information and Usage of Antibiotics in Poultry

To gain deeper insights into AMU practices, six additional questions were presented to farmers. Two of these questions required ‘Yes’ or ‘No’ responses, while the others utilized different categorical options to evaluate farmers' approaches to AMU.

### 2.11 Data Management and Analysis

Data collected through face‐to‐face interviews were initially recorded using paper‐based questionnaires and subsequently transferred to Microsoft Excel, a widely utilized software for data management and analysis. The software was used for data cleaning, organization and preliminary statistical evaluations. Descriptive statistics were employed to summarize the responses. The dataset included responses to closed‐ended questions, categorized as ‘yes’ or ‘no’ for knowledge and practices related to AMU and AMR. Attitude‐related questions were presented in two formats: One set offered three response options (‘yes,’ ‘no,’ or ‘no idea’), while another used a Likert scale (‘agree,’ ‘disagree,’ or ‘no idea’). Dissimilarities between groups were analysed using the Chi‐square test to evaluate associations between variables. Statistical significance was determined based on *p*‐values, with values below 0.05 considered significant, while those above 0.05 considered not significant. This approach ensured a robust analysis of the collected data, facilitating meaningful interpretation of the study's findings.

## 3 Results

### 3.1 Socio‐Demographic and Farming Characteristics of Respondents

This study surveyed 294 poultry farmers, with the majority being male (90.82%; *n* = 267), while 9.18% (*n* = 27) were female. Most farmers (71.42%; *n* = 210) were under 40 years old, including 17.69% (*n* = 52) aged 20–30 years and 53.74% (*n* = 158) aged 31–40 years. Educational attainment varied, with 44.90% (*n* = 132) having completed secondary education, 29.25% (*n* = 86) having primary education and only 6.46% (*n* = 19) holding a degree at the honours level or higher. A small proportion of respondents (3.74%; *n* = 11) had no formal education. Poultry farming was the primary occupation for 74.47% (*n* = 216) of respondents, while 26.53% (*n* = 78) were engaged in other professions. Experience in poultry farming was relatively high, with more than half of the farmers (60.54%; *n* = 178) had over 10 years of experience, while 18.03% (*n* = 53) having exceeding a more than a decade in the field. Regarding economic status, 62.24% (*n* = 183) reported a monthly family income of 15,000–30,000 BDT, while 14.63% (*n* = 43) earned less than 15,000 BDT and 23.13% (*n* = 68) earned over 30,000 BDT, reflecting a diverse economic background among participants. Farm characteristics indicated that 56.46% (*n* = 166) operated broiler farms, while 43.54% (*n* = 128) managed layer farms. Flock sizes varied, with 30.27% (*n* = 89) maintaining 1000–2000 birds, followed by 29.59% (*n* = 87) managing 500–1000 birds. Most farms (64.97%; *n* = 191) employed an all‐in‐all‐out production system, while 35.03% (*n* = 103) operated continuously. Nearly all farms (99.66%; *n* = 293) followed an intensive production system, with only one farm practicing extensive poultry farming system. Waste management practices also varied, with 53.74% (*n* = 158) utilizing poultry manure as fertilizer, 27.21% (*n* = 80) utilizing it as fish feed and 19.05% (*n* = 56) using it for both purposes. While 54.42% (*n* = 160) of farmers had received formal training in poultry management, adherence to hygiene practices was notably low. Only 6.46% (*n* = 19) of farmers wore protective clothing while working, whereas 93.54% (*n* = 275) worked in casual clothing, increasing the risk of pathogen exposure. Statistical analysis revealed significant associations between key demographic and farming variables, including educational qualification, farming experience, family income, flock size, production system and the use of protective uniforms (*p* = 0). A significant relationship was also found with farmer age (*p* = 0.007). Detailed data on these characteristics are presented in Table 1.

**TABLE 1:** Socio‐demographic and farming characteristics of poultry farmers.

	Overall *N* = 294	Broiler,166	Layer,128	
Variables	*n* (%)	*n* (%)	*n* (%)	*p*‐value
**Gender**				
Male	267 (90.82%)	146 (87.95%)	121 (94.53 %)	0.139
Female	27 (9.18%)	20 (12.05%)	7 (5.47%)	
**Age of farmer (year)**				
20–30	52 (17.69%)	40 (24.10%)	12 (9.37%)	0.007
31–40	158 (53.74%)	88 (53.01 %)	70 (54.69%)	
41–50	64 (21.77%)	31 (18.67%)	33 (25.78%)	
>50	20 (6.80%)	9 (5.42%)	11 (8.59%)	
**Educational qualification**				
Primary	86 (29.25%)	69 (41.57%)	17 (13.28%)	<0.0001
Secondary	132 (44.90%)	68 (40.96%)	64 (50%)	
Intermediate	46 (15.65%)	15 (9.04 %)	31 (24.22%)	
Honors or above	19 (6.46%)	6 (3.61%)	13 (10.16%)	
No formal education	11 (3.74%)	8 (4.82%)	3 (2.34%)	
**Main occupation**				
Poultry farming	216 (74.47%)	125 (75.30 %)	91 (71.09%)	0.418
Other than farming	78 (26.53%)	41 (24.70%)	37 (28.91%)	
**Experience (year)**				
0–5	63 (21.43%)	53 (31.93%)	10 (7.81%)	<0.0001
10	178 (60.54%)	100 (60.24%)	78 (60.94%)	
>10	53 (18.03%)	13 (7.83%)	40 (31.25%)	
**Family Income**				
<15000 BDT	43 (14.63%)	30 (18.07%)	13 (10.16 %)	<0.0001
15000–30000 BDT	183 (62.24%)	116 (69.88%)	67 (52.34%)	
>30000 BDT	68 (23.13%)	20 (12.05%)	48 (37.50%)	
**Flock size**				
<500	30 (10.20%)	30 (18.07 %)	0 (0%)	<0.0001
500–1000	87 (29.59%)	63 (37.95%)	24 (18.75%)	
1000–2000	89 (30.27%)	40 (24.10 %)	49 (38.28 %)	
2000–3000	53 (18.03%)	22 (13.25%)	31 (24.22%)	
3000–5000	27 (9.18%)	11 (6.63%)	16 (12.50%)	
>5000	16 (5.44%)	0 (0%)	8 (6.25%)	
**Training on poultry farming management**				
Yes	160 (54.42 %)	84 (50.60%)	76 (59.38%)	0.134
No	134 (45.58%)	82 (49.40%)	52 (40.63%)	
**Ranging style**				
Intensive system	293 (99.66 %)	166 (100%)	127 (99.22%)	<0.0001
Semi‐intensive	0 (0 %)	0 (0%)	0 (0%)	
Extensive	1 (.34%)	1 (0.60%)	0 (0 %)	
**Production system**				
All‐in‐all‐out	191 (64.97%)	122 (73.49 %)	69 (53.91 %)	<0.0001
Continuous	103 (35.03%)	44 (26.51%)	59 (46.09%)	
**Waste disposal ways**				
Use as fertilizer	158 (53.74%)	91 (54.82 %)	67 (52.34%)	0.914
Use as fish feed	80 (27.21%)	44 (26.51%)	36 (28.13%)	
Both	56 (19.05%)	31 (18.67%)	25 (19.53%)	
**Wearing uniform**				
Yes	19 (6.46%)	3 (1.81 %)	16 (12.50%)	<0.0001
No	275 (93.54%)	163 (98.19%)	112 (87.50 %)	
**Leaving uniform**				
Yes	11 (3.74%)	5 (3.01%)	6 (4.69%)	0.453
No	283 (96.26%)	161 (96.99%)	122 (95.31 %)	

### 3.2 Knowledge of Poultry Farmers Regarding AMU and AMR

The knowledge of poultry farmers about AMU and AMR was assessed through 10 binary (yes/no) questions, as summarized in Table 2. The findings revealed substantial gaps in farmers’ understanding of AMU and resistance. Only 34.01% (*n* = 100) of respondents demonstrated any knowledge of antimicrobials, with broiler farmers exhibiting slightly better awareness (39.16%; *n* = 65) compared to layer farmers (27.34%; *n* = 35; *p* = 0.033). In addition, 73.13% (*n* = 215) of farmers were unaware of the authority responsible for issuing antimicrobial prescriptions, with this lack of knowledge being more pronounced among broiler farmers (82.53%; *n* = 137) than layer farmers (60.94%; *n* = 78; *p* < 0.001). Understanding of antimicrobial residues was particularly limited, with only 5.44% (*n* = 16) of farmers being aware of their presence, while 94.56% (*n* = 278) had no knowledge of the concept. Awareness of antibiotic withdrawal periods was also poor, with only 8.16% (*n* = 24) demonstrating an understanding—broiler farmers showed slightly higher awareness (10.84%; *n* = 18) than layer farmers (4.69%; *n* = 6; *p* = 0.049). When asked about the appropriateness of administering antibiotics without a veterinarian's prescription, 65.31% (*n* = 192) believed it was acceptable, with a significantly higher proportion among broiler farmers (73.49%; *n* = 122) compared to layer farmers (54.69%; *n* = 70; *p* = 0.001). Widespread misconceptions were observed regarding antibiotic efficacy. While 93.54% (*n* = 275) correctly identified antibiotics as effective against bacterial infections, 80.61% (*n* = 237) incorrectly believed they were also effective against viral infections. Alarmingly, only 8.84% (*n* = 26) of respondents recognized that misuse or overuse of antibiotics could contribute to AMR, indicating a critical gap in awareness. In addition, 97.28% (*n* = 286) believed that antibiotics should be administered to an entire flock if a single bird was sick, highlighting a lack of understanding of prudent AMU practices. These findings underscore the urgent need for targeted educational initiatives and awareness campaigns to address the significant knowledge gaps related to AMU and AMR among poultry farmers. Improving farmer education and implementing regulatory measures could play a vital role in promoting responsible antimicrobial stewardship in Bangladesh's poultry industry.

**TABLE 2:** Distribution of knowledge/awareness about antimicrobial usage (AMU) and antimicrobial resistant (AMR) among poultry farmers.

Variables	Overall *N* = 294	Broiler, 166	Layer, 128	*p*‐value
	*n* (%)	*n* (%)	*n* (%)	
**Do you have any idea about antimicrobials?**				
Yes	100 (34.01%)	65 (39.16%)	35 (27.34%)	0.033
No	194 (65.99%)	101 (60.84%)	93 (72.66%)	
**Do you know who has the authority to write a prescription?**				
Yes	79 (25.87%)	29 (17.47%)	50 (39.06%)	<0.0001
No	215 (73.13%)	137 (82.53%)	78 (60.94%)	
**Do you know about antimicrobial residue?**				
Yes	16 (5.44%)	8 (4.82%)	8 (6.25%)	0.592
No	278 (94.56%)	158 (95.18%)	120 (93.75%)	
**Antibiotics are required for all flocks, when one bird is sick?**				
Yes	286 (97.28%)	161 (96.99%)	125 (97.66%)	0.725
No	8 (2.72%)	5 (3.01%)	3 (2.34%)	
**Antibiotics can be administered to poultry without veterinarians' prescription?**				
Yes	192 (65.31%)	122 (73.49%)	70 (54.69%)	0.001
No	102 (34.69%)	44 (26.50%)	58 (45.31%)	
**Antibiotics can be administered for all disease?**				
Yes	195 (66.33%)	132 (79.52%)	63 (49.22%)	<0.0001
No	99 (33.67%)	34 (20.48%)	65 (50.78%)	
**Antibiotics are effective for bacterial diseases**				
Yes	275 (93.54%)	152 (91.57%)	123 (96.09%)	0.021
No	19 (6.46%)	14 (8.43%)	5 (3.91%)	
**Antibiotics are effective for viral diseases?**				
Yes	237 (80.61%)	130 (78.31%)	107 (83.59%)	0.253
No	57 (19.39%)	36 (21.69%)	21 (16.41%)	
**Do you have knowledge about antibiotics resistance which can be developed due to overuse or misuse of antibiotics?**				
Yes	26 (8.84%)	12 (7.23%)	14 (10.94%)	0.267
No	268 (91.16%)	154 (92.77%)	114 (89.06%)	
**Do you know about antibiotics withdrawal period?**				
Yes	24 (8.16%)	18 (10.84%)	6 (4.69%)	0.049
No	270 (91.84%)	148 (89.16%)	122 (95.31%)	

### 3.3 Attitudes of Poultry Farmers Toward AMU and AMR

The attitudes of poultry farmers toward AMU and AMR were assessed using nine structured questions, as summarized in Table 3. Among these, five questions allowed responses categorized as ‘Yes’, ‘No’ or ‘No idea,’ while the remaining four questions were assessed using a Likert‐type scale with responses categorized as ‘Agree’, ‘Disagree’, or ‘No idea’ to evaluate farmers’ perspectives on AMU and AMR. The majority of respondents (79.25%; *n* = 233) believed that antimicrobials were primarily intended for treatment, with broiler farmers (81.93%; *n* = 136) slightly more likely to hold this view than layer farmers (75.78%; *n* = 97; *p* = 0.095). A considerable knowledge gap was observed regarding the relationship between inappropriate antibiotic use and AMR. A large proportion (82.65%; *n* = 243) of respondents lacked awareness of this connection, while only 14.63% (*n* = 43) correctly acknowledged that misuse of antibiotics could contribute to AMR. When questioned about the public health significance of AMR, 92.52% (*n* = 272) of farmers reported having no knowledge on this issue, while only 6.46% (*n* = 19) disagreed with the statement that AMR was not a significant public health concern. This suggests a lack of understanding among poultry farmers regarding the potential consequences of antibiotic resistance on human health. Similarly, the vast majority (95.24%; *n* = 280) of farmers were unaware of the link between antibiotic use in poultry and the development of resistance, further highlighting the urgency of awareness programs. On the importance of accurate dosing, 51.02% (*n* = 150) of farmers agreed that precise antimicrobial doses should be administered, with layer farmers (60.16%; *n* = 77) significantly more likely to support this practice than broiler farmers (43.98%; *n* = 73; *p* = 0.012). This finding suggests that layer farmers may have greater concern for dosage precision due to the extended production cycle of laying hens compared to broilers.

**TABLE 3:** Attitudes of poultry famers towards antimicrobial usage (AMU) and antimicrobial resistant (AMR) among poultry farmers.

Variables	Overall *N* = 294	Broiler,166	Layer,128	*p*‐value
**Was the antimicrobials used for treatment?**				
Yes	233 (79.25%)	136 (81.93%)	97 (75.78%)	0.095
No	50 (17.01%)	22 (13.25%)	28 (21.88%)	
No idea	11 (3.74%)	8 (4.82%)	3 (2.34%)	
**Inappropriate use of antibiotics may cause AMR**				
Yes	43 (14.63%)	12 (7.23%)	31 (24.22%)	<0.0001
No	8 (2.72%)	5 (3.01%)	3 (2.34%)	
No Idea	243 (82.65%)	149 (89.76%)	94 (73.44%)	
**AMR is not significant for public health?**				
Agree	32 (10.88%)	20 (12.05%)	12 (9.38%)	0.573
Disagree	19 (6.46%)	9 (5.42%)	10 (7.81%)	
No idea	272 (92.52%)	137 (82.53%)	106 (82.81%)	
**Is there any relationship between antibiotic use in poultry and the development of resistance?**				
Agree	9 (3.06%)	3 (1.81%)	6 (4.69%)	0.361
Disagree	5 (1.70%)	3 (1.81%)	2 (1.56%)	
No idea	280 (95.24%)	160 (96.39%)	120 (93.75%)	
**An accurate dose of antimicrobials should be used in poultry?**				
Agree	150 (51.02%)	73 (43.98%)	77 (60.16%)	0.012
Disagree	7 (2.38%)	4 (2.41%)	3 (2.34%)	
No idea	137 (46.60%)	90 (54.22%)	47 (36.82%)	
**The herbal or medicinal drugs can be used as alternatives to antimicrobials?**				
Agree	20 (6.80%)	15 (9.04%)	5 (3.91%)	0.03
Disagree	9 (3.06%)	8 (4.82%)	1 (0.78%)	
No idea	265 (90.14%)	143 (86.14%)	122 (95.31%)	

However, knowledge of herbal or medicinal alternatives to antibiotics was minimal, with only 6.80% (*n* = 20) agreeing that such alternatives could be used as substitutes for antimicrobials, while 90.14% (*n* = 265) admitted having no knowledge of them. This lack of awareness could be attributed to insufficient exposure to alternative veterinary practices or a strong dependence on conventional antibiotic‐based treatments. These findings highlight significant gaps in farmers’ awareness and attitudes toward AMU and AMR. The widespread misconceptions and lack of understanding emphasize the urgent need for targeted education initiatives, awareness campaigns and regulatory interventions to promote responsible AMU in poultry farming.

### 3.4 AMU Practices Among Poultry Farmers

AMU practices among poultry farmers, including those managing broiler and layer farms, were assessed through six specific questions. The responses were categorized as ‘Yes’ or ‘No,’ with a detailed summary presented in Table 4. A significant proportion of farmers (69.73%; *n* = 205) reported administering antibiotics to sick birds without consulting a veterinarian. This practice was more prevalent among layer farmers (78.13%; *n* = 100) than broiler farmers (63.25%; *n* = 105, *p* < 0.001), indicating a greater reliance on self‐prescribed antibiotic use among layer farmers. In addition, only 34.01% (*n* = 100) of farmers reported reading the antibiotic prospectus before use, with layer farmers (42.97%; *n* = 55) being more likely to do so than broiler farmers (27.11%; *n* = 45, *p* = 0.004). This discrepancy may be attributed to differences in management practices between layer and broiler farms, where longer production cycles in layer farms may encourage greater attention to medication details. The use of antibiotics during the brooding period was reported by 62.24% (*n* = 183) of farmers, with no significant difference between broiler (60.24%; *n* = 100) and layer farmers (64.84%; *n* = 83, *p* = 0.419). This widespread use of antibiotics in young birds suggests a potential over‐reliance on antimicrobials for disease prevention, raising concerns about early exposure to antibiotics and its implications for AMR. A substantial proportion of farmers (96.60%; *n* = 284) reported using antimicrobials as a preventive measure, with no significant differences between broiler and layer farmers. However, only 6.46% (*n* = 19) of farmers used antimicrobials exclusively to treat sick birds, with broiler farmers (2.41%; *n* = 4) being significantly less likely to adopt this practice compared to layer farmers (11.72%; *n* = 15; *p* = 0.005). Regarding the use of antimicrobials as growth promoters, 39.46% (*n* = 116) of farmers admitted to engaging in this practice, with a notable difference between broiler (56.63%; *n* = 94) and layer farmers (17.19%; *n* = 22; *p* < 0.001). This finding indicates that broiler farmers are more inclined to use antibiotics to promote growth, possibly due to the shorter production cycle and higher market demand for rapid weight gain in broilers. A critical gap in veterinary guidance was also evident, as only 12.59% (*n* = 37) of farmers had received veterinary guidance on the withdrawal period, with layer farmers (18.75%; *n* = 24) more likely to receive such information than broiler farmers (7.83%; *n* = 13, *p* = 0.005). This lack of awareness contributes to the risk of antimicrobial residues in poultry products, posing potential public health concerns. Furthermore, most farmers (90.48%; *n* = 266) reported discontinuing antibiotics once birds appeared to recover rather than completing the prescribed course, a practice slightly more common among broiler farmers (93.37%; *n* = 155) than layer farmers (86.72%; *n* = 111, *p* = 0.054). In addition, 84.35% (*n* = 248) of farmers admitted to selling broilers or eggs during or shortly after antibiotic use, with no significant difference between broiler (81.93%; *n* = 136) and layer farmers (87.50%; *n* = 112, *p* = 0.188). These findings highlight critical concerns regarding AMU practices, including the frequent lack of veterinary consultation, incomplete antibiotic courses and disregard for withdrawal periods. Such behaviours elevate the risk of antimicrobial residues in poultry products, exacerbating the growing threat of AMR.

**TABLE 4:** Practice of antimicrobials by poultry farmers towards antimicrobial usage (AMU) and antimicrobial resistance (AMR).

Variables	Overall *N* = 294	Broiler,166	Layer,128	*p*‐value
	*n* (%)	*n* (%)	*n* (%)	
**Use antibiotics before consulting a vet in case sick bird?**				
Yes	205 (69.73%)	105 (63.25%)	100 (78.13%)	<0.0001
No	89 (30.27%)	61 (36.75%)	28 (21.88%)	
**Do you read the prospectus before using antibiotic?**				
Yes	100 (34.01%)	45 (27.11%)	55 (42.97%)	0.004
No	194 (65.97%)	121 (72.89%)	73 (57.03%)	
**Do you use any antibiotics during brooding period?**				
Yes	183 (62.24%)	100 (60.24%)	83 (64.84%)	0.419
No	111 (37.76%)	66 (39.76%)	45 (35.16%)	
**Did you use antimicrobials to sick Birds only?**				
Yes	19 (6.46%)	4 (2.41%)	15 (11.72%)	0.005
No	271 (92.18%)	159 (95.78%)	112 (87.50%)	
No idea	4 (1.36%)	3 (1.81%)	1 (0.78%)	
**Did you use antimicrobials to prevent disease?**				
Yes	284 (96.60%)	161 (96.99%)	123 (96.09%)	0.392
No	4 (1.36%)	1 (0.60%)	3 (2.34%)	
No idea	6 (2.04%)	4 (2.41%)	2 (1.56%)	
**Did you use antimicrobials as growth promoters?**				
Yes	116 (39.46%)	94 (56.63%)	22 (17.19%)	<0.0001
No	61 (20.75%)	22 (13.25%)	39 (30.47%)	
No Idea	117 (39.80%)	51 (30.72%)	67 (52.34%)	
**Did you get information from the vet about withdrawal period?**				
Yes	37 (12.59%)	13 (7.83%)	24 (18.75%)	0.005
No	257 (87.41%)	153 (92.17%)	104 (81.25%)	
**Do you stop the application of the dose when birds feel better?**				
Yes	266 (90.48%)	155 (93.37%)	111 (86.72%)	0.054
No	28 (9.52%)	11 (6.63%)	17 (13.28%)	
**Selling eggs and broilers during and after using drugs?**				
Yes	248 (84.35%)	136 (81.93%)	112 (87.50%)	0.188
No	46 (15.65%)	30 (18.07%)	16 (12.50%)	

### 3.5 Information and Usage of Antibiotics in Poultry

To gain deeper insights into AMU practices among poultry farmers, six additional questions were posed, with responses categorized as ‘Yes’ or ‘No’ or grouped into relevant categories. The findings, summarized in Table 5, reveal that antimicrobials were used on nearly all surveyed farms (98.64%; *n* = 290), with comparable usage rates between broiler (97.59%; *n* = 162) and layer farmers (98.44%; *n* = 126, *p* = 0.034). Farmers obtained information on AMU from a variety of sources, with veterinarians and dealers being the primary sources of information on antimicrobials. However, the reliance on veterinary consultation differed significantly between broiler and layer farmers. Overall, 50.34% (*n* = 148) of farmers reported receiving guidance from veterinarians, though this varied significantly by farm type—only 24.10% (*n* = 40) of broiler farmers consulted veterinarians compared to 84.38% (*n* = 108) of layer farmers (*p* < 0.001). Conversely, broiler farmers relied more on dealers (37.35%; *n* = 62) than layer farmers (9.38%; *n* = 12). Regarding prescriptions, 55.44% (*n* = 163) of farmers obtained them from veterinarians, with a significantly higher proportion among layer farmers (76.56%; *n* = 98) compared to broiler farmers (39.16%; *n* = 65, *p* < 0.001). The remaining 44.56% (*n* = 131) of farmers either self‐prescribed antimicrobials or relied on non‐veterinary advice, reflecting a potential gap in antibiotic stewardship and regulatory oversight. Most farmers sourced antibiotics from pharmacies (48.98%; *n* = 144) or distributors (44.90%; *n* = 132). Broiler farmers more frequently purchased from distributors (53.01%; *n* = 88) than pharmacies (42.17%; *n* = 70), whereas layer farmers primarily relied on pharmacies (57.81%; *n* = 74, *p* = 0.006). Water was the predominant method of antimicrobial administration, used by 96.94% (*n* = 285) of farmers. This practice was nearly universal among broiler farmers (99.40%; *n* = 165) compared to layer farmers (93.75%; *n* = 120, *p* = 0.004). Only a small percentage (3.06%; *n* = 9) of farmers administered antibiotics via feed. Most farmers (90.48%; *n* = 266) reported using antibiotics once per month, while 9.52% (*n* = 28) administered them more frequently. Broiler farmers (12.05%; *n* = 20) were more likely to use antibiotics multiple times per month than layer farmers (6.25%; *n* = 8, *p* = 0.087), though the difference was not statistically significant. These findings highlight the pervasive and often unregulated use of antimicrobials in poultry farming, characterized by reliance on non‐veterinary sources, inconsistent prescription practices and widespread administration via water. Such practices may exacerbate the risk of AMR. Strengthening regulatory frameworks and enhancing farmer education are crucial steps toward mitigating these risks and promoting responsible AMU in poultry production.

**TABLE 5:** Information and usage of antibiotics for poultry.

Variables	Overall *N* = 294	Broiler,166	Layer,128	*p*‐value
	*n* (%)	*n* (%)	*n* (%)	
**Have you given antibiotics to your poultry?**				
Yes	290 (98.64%)	162 (97.59%)	126 (98.44%)	0.034
No	4 (1.36%)	4 (2.41%)	2 (1.56%)	
**Source of information about antimicrobials**				
Veterinarian	148 (50.34%)	40 (24.01%)	108 (84.38%)	< 0.0001
Dealers	74 (25.17%)	62 (37.35%)	12 (9.38%)	
Himself	43 (14.63%)	35 (21.8%)	8 (6.25%)	
Friends	29 (9.86%)	29 (17.47%)	0 (0%)	
**Did veterinarian prescribe the antimicrobials?**				
Yes	163 (55.44%)	65 (39.16%)	98 (76.56%)	< 0.0001
No	131 (44.56%)	101 (60.84%)	30 (23.44%)	
**From where did you purchase the antimicrobials?**				
Pharmacy	144 (48.98%)	70 (42.17%)	74 (57.81%)	0.006
Distributer	132 (44.90%)	88 (53.01%)	44 (34.38%)	
Company	18 (6.12%)	8 (4.82%)	10 (7.81%)	
**Route of antimicrobial administrations**				
Through water	285 (96.94%)	165 (99.40%)	120 (93.75%)	0.004
Through feed	9 (3.06%)	1 (0.60%)	8 (6.25%)	
**Antimicrobials given per month**				
Once	266 (90.48%)	146 (87.95%)	120 (93.75%)	0.087
More than once	28 (9.52%)	20 (12.05%)	8 (6.25%)	

### 3.6 Preference and Frequency of Antimicrobial Usage Among Poultry Farms

**FIGURE 2:**
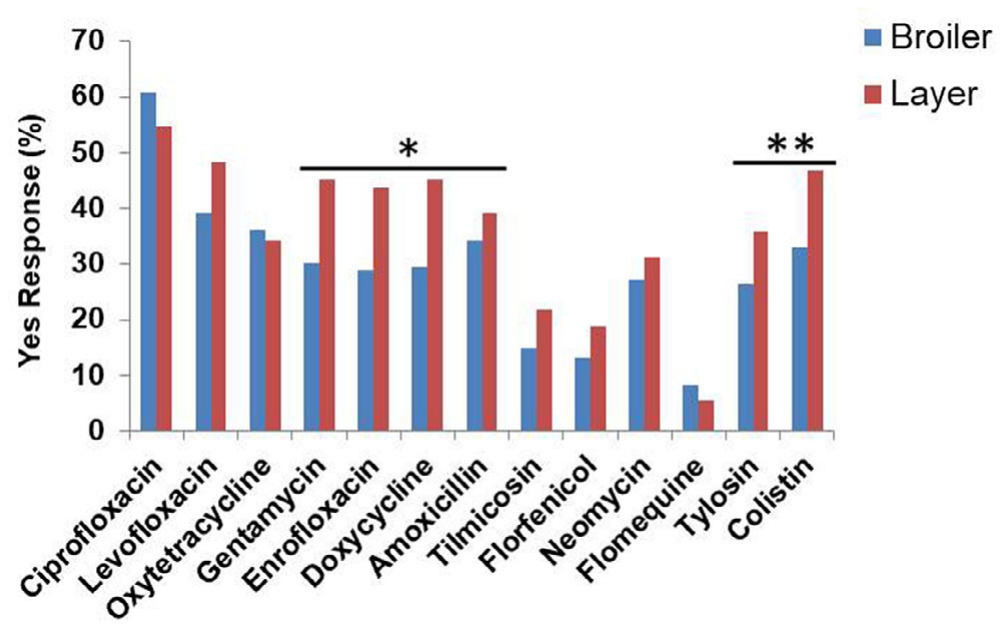
Frequently used antimicrobials among broiler and layer farmers. This bar chart illustrates the percentage of yes responses regarding the use of various antimicrobials among broiler (blue) and layer (red) farmers. Antimicrobials assessed include Ciprofloxacin, Levofloxacin, Oxytetracycline, Gentamycin, Enrofloxacin, Doxycycline, Amoxicillin, Tilmicosin, Florfenicol, Neomycin, Florfenicol, Tylosin and Colistin. Statistical significance was determined using the chi‐square (*χ* ^2^) test, with * and ** indicating *p* < 0.05.

The preferences and frequencies of AMU among broiler and layer farmers were assessed to identify common antibiotics used in poultry farming and their administration patterns. The findings, summarized in Table 6, indicate that multiple antibiotics were frequently used at various production stages, with broiler farms administering antimicrobials at least three key phases of the production cycle. Across all surveyed farms, 13 different antibiotics (identified by their generic names) were reported. Ciprofloxacin was the most frequently used antimicrobial, with 60.84% of broiler farmers and 54.69% of layer farmers reporting its use, though the difference was not statistically significant (*p* = 0.289). Colistin, another widely used antibiotic, exhibited a statistically significant difference in usage rates between the two farming systems, with 46.88% of layer farmers administering it compared to 33.13% of broiler farmers (*p* = 0.017). Other commonly used antibiotics included gentamycin, enrofloxacin, doxycycline and amoxicillin, with variations in usage patterns between broiler and layer farms. These findings underscore the widespread reliance on antimicrobials in poultry production, often in the absence of standardized guidelines or stringent oversight. The frequent use of ciprofloxacin and colistin—both critical in human medicine—raises significant concerns regarding the emergence and spread of AMR. Targeted antimicrobial stewardship strategies and stricter regulatory frameworks are urgently needed to mitigate this growing public health threat (Figure 2).

**TABLE 6:** Frequently used antimicrobials among broiler and layer farmers.

	Overall *N* = 294	Broiler,166	Layer,128	
Variables	*n* (%)	*n* (%)	*n* (%)	*p*‐value
**Ciprofloxacin**				
Yes	171 (58.16%)	101 (60.84%)	70 (54.69%)	**0.289**
No	123 (41.84%)	65 (39.16%)	58 (45.31%)	
**Levofloxacin**				
Yes	127 (43.20%)	65 (39.16%)	62 (48.44%)	**0.111**
No	167 (56.80%)	101 (60.84%)	66 (51.56%)	
**Oxytetracycline**				
Yes	104 (35.37%)	60 (36.14%)	44 (34.38%)	**0.753**
No	190 (64.63%)	106 (63.86%)	84 (65.63%)	
**Gentamycin**				
Yes	108 (36.73%)	50 (30.12%)	58 (45.31%)	**0.007**
No	186 (63.27%)	116 (69.88%)	70 (54.69%)	
**Enrofloxacin**				
Yes	104 (35.37%)	48 (28.92%)	56 (43.75%)	**0.007**
No	190 (64.63%)	118 (71.08%)	72 (56.25%)	
**Doxycycline**				
Yes	107 (36.39%)	49 (29.52%)	58 (45.31%)	**0.005**
No	187 (63.61%)	117 (70.48%)	70 (54.69%)	
**Amoxicillin**				
Yes	107 (36.39%)	57 (34.34%)	50 (39.06%)	**0.005**
No	187 (63.61%)	109 (65.66%)	78 (60.94%)	
**Tilmicosin**				
Yes	53 (18.03%)	25 (15.06%)	28 (21.88%)	**0.132**
No	241 (81.97%)	141 (84.94%)	100 (78.13%)	
**Florfenicol**				
Yes	46 (15.65%)	22 (13.25%)	24 (18.75%)	**0.198**
No	248 (84.35%)	144 (86.75%)	104 (81.25%)	
**Neomycin**				
Yes	85 (28.91%)	45 (27.11%)	40 (31.25%)	**0.437**
No	209 (71.09%)	121 (72.89%)	88 (68.75%)	
**Flomequine**				
Yes	21 (7.14%)	14 (8.43%)	7 (5.47%)	**0.437**
No	273 (91.86%)	152 (91.57%)	121 (94.53%)	
**Tylosin**				
Yes	91 (30.95%)	44 (26.51%)	46 (35.94%)	**0.082**
No	203 (79.05%)	122 (73.89%)	82 (64.06%)	
**Colistin**				
Yes	115 (39.12%)	55 (33.13%)	60 (46.88%)	**0.017**
No	179 (60.88%)	111 (66.86%)	68 (53.13%)	

## 4 Discussion

AMU and AMR have emerged as critical global health challenges, posing significant risks to both human and animal populations. The misuse and overuse of antibiotics in poultry farming, driven by limited awareness, economic constraints and improper practices, have accelerated the emergence of AMR. This study examined the KAP of broiler and layer farmers in Bangladesh regarding AMU and AMR. The findings provide valuable insights into the underlying factors influencing AMU practices and underscore the urgent need for targeted interventions to mitigate the risk of AMR within the poultry sector.

The study revealed that the majority of poultry farmers were under 40 years old, with primary or secondary education being the most common level of formal schooling. This finding aligns with previous research indicating that younger individuals dominate the poultry farming sector in Bangladesh (M. M. Hassan et al. 2021). Poultry farming served as the primary livelihood for most respondents, particularly in rural areas where alternative employment opportunities remain scarce (DLS 2023). Although the majority of farmers had over a decade of experience, gaps in formal education and training on AMU persisted. Such deficiencies in knowledge and training may hinder informed decision‐making regarding AMU, reinforcing the need for structured educational programs (McKernan et al. 2021; Regan et al. 2023).

A key finding of this study was the substantial influence of poultry dealers on AMU decisions. Dealers played a pivotal role in distributing antibiotics and advising farmers on their use, with 25.17% of respondents relying on them as their primary source of information (Begum et al. 2013; M. M. Hassan et al. 2021; Masud et al. 2020; Poudel et al. 2024). This strong dependence on dealers stems from the financial and logistical support they provide, including feed, chicks, and medicines on credit. However, this arrangement raises concerns regarding conflicts of interest, as dealers may prioritize sales over the prudent use of antimicrobials. Alarmingly, 44.56% of farmers bypassed veterinary consultations entirely, primarily due to cost constraints, limited access to veterinarians in rural areas, and a general lack of awareness about the importance of professional guidance (M. M. Hassan et al. 2021; Masud et al. 2020; Siddiky et al. 2022).

Several improper AMU practices contributing to AMR were identified. A significant proportion of farmers administered antibiotics during the brooding period, often without clinical justification (Islam et al. 2022). Critically important antimicrobials, such as ciprofloxacin and colistin, were widely used as preventive measures rather than for treating diagnosed infections (Roess et al. 2013; World Health Organization 2021; Habiba et al. 2023). Furthermore, adherence to antibiotic withdrawal periods was notably low, with only 12.59% of farmers receiving veterinary guidance on withdrawal protocols (Siddiky et al. 2022). Such inadequate compliance with withdrawal periods poses a substantial risk of antimicrobial residues in poultry products, increasing the likelihood of human exposure to resistant bacteria through food consumption.

Biosecurity measures were inadequately implemented, particularly among small‐scale farms (Shaparan 2022). Many farmers relied on antibiotics to compensate for suboptimal hygiene practices, rather than addressing fundamental issues related to farm management and sanitation. This over‐reliance on antimicrobials as a substitute for proper biosecurity underscores the urgent need for targeted training programs to promote responsible AMU (McKernan et al. 2021). However, a key limitation of this study is its primary focus on AMU practices, with limited assessment of biosecurity and disease prevention strategies; future research should integrate these aspects to provide a more comprehensive understanding of AMR drivers in poultry farming. Despite existing regulations mandating prescriptions for antibiotic purchases, over‐the‐counter sales remain widespread due to weak enforcement mechanisms (Jhora 2021).

Despite existing regulations mandating prescriptions for antibiotic purchases, over‐the‐counter sales remain widespread due to weak enforcement mechanisms. The Ministry of Health and Family Welfare's NAP (2017–2022) aimed to combat AMR, yet its implementation has been hampered by insufficient resources and poor coordination among stakeholders (Jhora 2021). Strengthening regulatory oversight, enhancing enforcement mechanisms and establishing antimicrobial stewardship programs are essential to curbing misuse and mitigating the AMR crisis.

## 5 Conclusion and Future Recommendations

This study provides critical insights into the KAP of poultry farmers regarding AMU and AMR in Bangladesh. The findings highlight the significant influence of socioeconomic factors, including education levels, farming experience and financial constraints, on AMU practices and farmers’ understanding of AMR. A major concern is the widespread reliance on antibiotic dealers for guidance, limited veterinary consultation and poor adherence to withdrawal periods. In addition, the unregulated use of critically important antimicrobials, such as ciprofloxacin and colistin, without appropriate indications poses severe public health risks. Addressing these challenges requires a multifaceted approach that integrates farmer education, stricter regulatory enforcement, improved veterinary services and alternative disease prevention strategies. To mitigate AMR and ensure sustainable antimicrobial practices in poultry farming, the following key recommendations should be prioritized:

### 5.1 Enhancing Education and Training

Raising awareness about responsible AMU is crucial for improving farmers’ decision‐making and reducing unnecessary dependency on antibiotics (McKernan et al. 2021; Ting et al. 2022; Regan et al. 2023). Training programs should focus on proper antibiotic use, adherence to withdrawal periods and alternative disease management strategies, including biosecurity, vaccination and probiotics (Haque et al. 2020). The Department of Livestock Services (DLS), academic institutions and NGOs such as BRAC should lead these initiatives, with support from the Ministry of Fisheries and Livestock (MoFL), FAO, WHO and donor agencies like USAID (WHO 2016). A collaborative, multi‐stakeholder approach will ensure sustainable knowledge dissemination.

### 5.2 Strengthening Veterinary Services

Expanding access to veterinary care, particularly in rural areas, is essential for promoting responsible AMU in poultry farming (Siddiky et al. 2022). A major challenge is the shortage of qualified veterinarians, which has led many farmers to rely on untrained dealers for antibiotic recommendations and disease management (44.56% bypassed veterinary consultation) (Islam et al. 2022; M. M. Hassan et al. 2021; Siddiky et al. 2022). This gap in veterinary support is exacerbated by weak enforcement of AMU regulations, further increasing the risk of misuse and overuse of antimicrobials (Jhora 2021). Deploying veterinarians with government incentives, establishing mobile veterinary units and integrating telemedicine services can bridge this gap. The Bangladesh Veterinary Association (BVA) should play a leading role in advocating for enhanced veterinary infrastructure, expanding training programs and rural service expansion.

### 5.3 Implementing Antimicrobial Stewardship Programs

The establishment of a national antimicrobial stewardship program is essential for regulating AMU in agriculture and addressing the growing threat of AMR (WHO 2016). Such a program should focus on educating farmers, enforcing prescription regulations and promoting judicious antibiotic use to ensure sustainable and responsible AMU practices in poultry farming. In addition, comprehensive and standardized guidelines for AMU in poultry farming should be developed, implemented and strictly monitored to ensure adherence to responsible antibiotic practices (WHO 2016).

### 5.4 Promoting Alternative Solutions

Encouraging the adoption of non‐antibiotic alternatives, such as vaccination, probiotics, herbal treatments, and improved hygiene, can reduce dependency on antimicrobials (Haque et al. 2020). Farmers should receive training on integrating these alternatives into poultry production, supported by government incentives and research‐based recommendations (Poudel et al. 2024).

### 5.5 Strengthening Regulatory Measures and Surveillance

Stricter enforcement of existing AMU regulations is necessary to curb the over‐the‐counter sale of antibiotics (48.98% purchased without consultation) and prevent their misuse in poultry farming (Jhora 2021). Ensuring that antibiotics are dispensed only with veterinary prescriptions will help regulate their use and promote responsible administration. In addition, the establishment of robust AMU surveillance systems is critical for monitoring compliance, tracking antibiotic consumption patterns and identifying emerging resistance trends (Go'chez et al. 2019; WHO 2016). Strengthening these regulatory frameworks will play a key role in mitigating AMR and ensuring sustainable poultry production.

### 5.6 Supporting Small‐Scale and Low‐Income Farmers

Financial support for small‐scale farmers can reduce their reliance on dealers for credit‐based antibiotic purchases (Masud et al. 2020). Governments and NGOs should offer low‐interest loans, subsidies or financial assistance programs to help farmers implement better biosecurity measures and adopt sustainable AMU practices (Begum et al. 2013; Faroque et al. 2023). Ensuring access to financial resources will enable farmers to implement better disease prevention strategies, reducing unnecessary antibiotic reliance and promoting responsible poultry farming practices.

### 5.7 Fostering Cross‐Sector Collaboration

Effectively combating AMR requires coordinated efforts across government agencies, international organizations (FAO, WHO, OIE) and industry stakeholders (WHO 2016). Strengthening public–private partnerships can drive awareness campaigns, research collaborations and policy development, ensuring a unified and strategic response to AMR challenges in poultry farming. A collaborative approach will enhance regulatory enforcement, knowledge sharing and sustainable antimicrobial stewardship, contributing to long‐term solutions for responsible antibiotic use in poultry farming (Al Masud et al. 2020).

## Author Contributions

**Zohir Raihan**: data curation, formal analysis, investigation, validation, writing – original draft, writing – review and editing, methodology. **Md. Nasir Uddin**: data curation, formal analysis, investigation, methodology, validation, writing – original draft, writing – review and editing. **Suman Das Gupta**: writing – review and editing. **M. Sawkat Anwer**: writing – review and editing. **Subir Sarker**: writing – review and editing. **Md. Hakimul Haque**: conceptualization, formal analysis, methodology, supervision, validation, writing – original draft, writing – review and editing, software, data curation, funding acquisition, investigation, visualization, project administration, resources.

## Funding

The authors have nothing to report.

## Conflicts of Interest

The authors declare no conflicts of interest.

## Data Availability

The data that support the findings of this study are available from the corresponding author upon reasonable request.
